# Comment on Loddé et al. Does Decreased Diffusing Capacity of the Lungs for Carbon Monoxide Constitute a Risk of Decompression Sickness in Occupational Divers? *Int. J. Environ. Res. Public Health* 2023, *20*, 6516

**DOI:** 10.3390/ijerph21111486

**Published:** 2024-11-08

**Authors:** Neal W. Pollock, Mikael Gennser, S. Lesley Blogg, Jan Risberg

**Affiliations:** 1 Department of Kinesiology, Université Laval, Québec, QC G1V 0A6, Canada; 2 Service de Médecine Hyperbare, Centre de Médecine de Plongée du Québec, Hôtel-Dieu de Lévis, Lévis, QC G6V 3Z1, Canada; 3 Division of Environmental Physiology, Royal Institute of Technology, 114 28 Stockholm, Sweden; gennser@kth.se; 4 SLB Consulting, Home Park Barn, Newbiggin-on-Lune CA17 4NX, UK; slblogg@hotmail.com; 5 NUI, 245 Bergen, Norway; jri@nui.no

**Keywords:** diving, ultrasound, decompression, safety, physiology, science publication

## Abstract

This letter addresses errors in the statistical analysis found in a paper addressing pulmonary diffusing capacity and decompression sickness. Our re-analysis could not confirm any of the significant statistical contrasts described for the bubble data, invalidating the speculation on the relationships between bubble scores and decompression sickness.

## 1. Correcting the Research Record—Concerns Over a Report of DLCO and Decompression Stress

This letter addresses concerns with a recent report describing a pilot effort to investigate the association between pulmonary diffusing capacity and the risk of decompression sickness [1]. The research question was of interest, but we found issues in the paper regarding unclear reporting and what appear to be erroneous statistical findings concerning the venous gas emboli (VGE) data. We do not address any of the non-VGE-related measures taken. We approached the authors both directly and through the journal with requests to examine the source data but did not receive a response. This is a concern given the data availability statement in the manuscript that the datasets used are available from the corresponding author upon reasonable request. Given the lack of access, we have been compelled to provide our feedback based on the pooled data presented in the manuscript.

The report describes a group of 15 divers who were examined with pulmonary function tests, including measurement of their pulmonary carbon monoxide diffusing capacity (DLCO), before experimental dives. The authors allocated subjects with DLCO < 85% of expected values to the “DLCO group” and subjects with DLCO ≥ 85% of expected values to the “Control group”. The source of the expected values was not provided.

All divers completed a controlled, immersed pool dive breathing air to 20 msw for 40 min, followed by a 3 min decompression stop at 3 msw during surfacing. Two-dimensional echocardiographic scans for VGE in the heart were conducted at 30 and 60 min post-dive, with measures recorded at rest and after deep knee bend provocations. The VGE scores were categorized according to the Eftedal–Brubakk scale and reportedly analyzed with chi square tests, with VGE scores pooled into categories of low grade (1–2) and high grade (3–5).

The authors reported that the VGE grades were significantly higher among divers in the DLCO group for rest and post-provocation at both time points measured. They also reported a significant delay in the time to peak VGE in the DLCO group compared to the control group (60 min vs. 30 min post-dive) both at rest and post-provocation, as well as an “almost significant” difference for the resting measures at 30 min. This led them to conclude that decreased DLCO is associated with a higher VGE grade with delayed kinetics. They ultimately hypothesized that lower DLCO values might be associated with a lower threshold for decompression sickness.

The text and table and figure captions offer conflicting descriptions of the subject counts; either 9 or 6 for the control group and either 6 or 9 for the DLCO group (cf caption and table heading, Table 1). Figure 1 (maximal VGE scores) appears to depict nine subjects in the Control group and seven subjects in the compromised DLCO group, the latter exceeding the reported count total. Figure 2a–d consistently depicted nine subjects in the control group and six in the DLCO group.

The primary problem is that the statistical significance reported for all contrasts (rest and post-provocation at 30 and 60 min post-dive) cannot be achieved with the distributions reported. Our re-analysis of the data yielded no significant differences in any of the stated contrasts. We extracted the values from Figure 1 and analyzed the data in a manner described by the authors, comparing “low grades” (pooled grade 1 and 2 scores and then pooled 0–2 scores) against “high grades” (pooled grade 3–5 scores) for the “Control” and “Reduced DLCO” groups. We ran statistics using both chi square and Fisher Exact tests. None of the combinations or tests yielded statistically significant differences (accepted as *p* < 0.05) in the contrasts. This invalidates the comments made on group differences in peak VGE scores and latency to peak bubble formation. The speculation as to whether an impaired pulmonary diffusing capacity might differentially affect inert gas elimination but not uptake is similarly not supported. The comments made in the limitation subsection on the peak of bubble formation are also problematic. The authors speculate that the peak in VGE may have occurred after the 60 min sample, but it is just as possible that the peak occurred before or around the measured points. It is inappropriate to speculate on patterns for measures not taken. The relevant limitation is that measuring bubble scores only twice post-dive failed to meet the recommended practice of sampling every 15–20 min for two hours to investigate decompression stress [2].

The collective effect of the shortcomings described here is an inability to trust the interpretations or conclusions of the work. More carefully designed, conducted, and analyzed research is needed to address the open questions.
